# Efficacy of a Novel Herbal Formulation (F2) on the Management of Obesity: *In Vitro* and *In Vivo* Study

**DOI:** 10.1155/2021/8854915

**Published:** 2021-02-08

**Authors:** Prakash Raj Pandeya, Ramakanta Lamichhane, Kyung-Hee Lee, Gopal Lamichhane, Se-Gun Kim, Hyun-Ju Jung

**Affiliations:** ^1^Department of Oriental Pharmacy and Wonkwang-Oriental Medicines Research Institute, Wonkwang University, Sinyong-Dong, Iksan 570-749, Republic of Korea; ^2^Department of Agricultural Biology, National Academy of Agricultural Science, Rural Development Administration, Wanju 566-851, Republic of Korea

## Abstract

**Background:**

Currently, obesity and its comorbidities have become a serious threat to human health necessitating urgent development of safe and effective therapy for their management.

**Materials and Methods:**

In this research, a novel polyherbal formulation (F2) was prepared by mixing specific proportions of royal jelly and lemon juice with ethanol extracts of *Orostachys japonicus*, *Rhus verniciflua*, and *Geranium thunbergii*. The antioxidant activity was assessed using DPPH and ABTS assay methods. The antiobesity potential of the F2 was assessed *in vitro* using 3T3-L1 fibroblast and *in vivo* using a high-fat diet (HFD) fed C57BL/6J mice model. F2 was administered in mice at the dose of 23 mg/kg and 46 mg/kg, twice daily by oral gavage. A well-accepted antiobesity agent, *Garcinia cambogia* (GC), at 200 mg/kg was used as a positive control.

**Results:**

F2 was observed to exhibit synergistic antiadipogenic activity in 3T3-L1 cells. This inhibition was reinforced by the downregulation of specific adipogenic transcription factors. Furthermore, F2 was also found to reduce mice body weight gain, food efficiency ratio, fasting blood glucose level, fat deposition into the liver, and mass of white adipose tissue. F2 also played a role in the excretion of fat consumed by the mice. For most of the assays performed, the F2 (46 mg/kg) was comparable to the positive control GC (200 mg/kg). In addition, potential and synergistic antioxidant activity was observed on F2.

**Conclusion:**

The results revealed that the formulation F2 exhibited potential antiobesity activity through the inhibition of adipocyte differentiation, dietary fat absorption, and reduction of free fatty acids deposition in tissues.

## 1. Introduction

According to World Health Organization (WHO), obesity is the accumulation of abnormal or excessive fat into the body to the extent that may impair health condition. Based on body mass index (BMI), an adult is considered overweight if the BMI is greater than or equal to 25 and becomes obese when BMI reaches more than 30 [1]. About 40% of the world's current population is suffering from overweight or obesity and is still in an increasing pattern [2]. Besides, childhood obesity is becoming a serious issue in the present time. WHO reports say 42 million children under 5 years of age were overweight or obese in 2016 [3]. Obesity is characterized by the increased size of adipocytes due to accumulation of lipids (hypertrophy), and/or increased number of adipocytes due to over-differentiation of preadipocytes into adipocytes (hyperplasia) under the influence of appropriate nutrition and hormonal release [4–6]. Obesity is associated with multiple metabolic disorders such as type 2 diabetes, cardiovascular diseases, fatty liver disease, mental disorders, and even certain cancers. Primarily, obesity may be controlled or prevented by nonpharmaceutical practices such as taking of restricted-calorie diet, physical exercise, and changing eating behavior. If the nonpharmaceutical practice does not give proper results, pharmaceutical interventions may be suggested [7]. Due to having more adverse effects, high cost, and a physical dependency of the pharmaceutical medicines, the uses of herbal medicine are increasing globally for the management of overweight and obesity [8]. Still, individual plants or phytochemical constituents may not be sufficient to achieve the desired therapeutic effect. A combination of multiple herbs may give a better therapeutic effect with reduced toxicity [9].

*Orostachys japonicus* (OJ) A. Berger (Crassulaceae) is a Korean medicinal plant. Traditionally, OJ has been used in the treatment and/or prevention of gastric ulcers, hepatitis, hemorrhoids, hematemesis, and cancer [10, 11]. The phytochemical composition of OJ includes triterpene, sterols, and flavonoids [11]. Previous studies revealed that OJ has shown potential antioxidant, hypoglycaemic, anticancer, anti-HIV-1 protease, antiulcerogenic, analgesic, immunostimulatory, and hepatoprotective effects [10–12]. Antiadipogenic and antiobesity effects of OJ were also described previously in various in vitro and in vivo studies [10, 13, 14].

*Rhus verniciflua* (RV) Stokes (Anacardiaceae) has been used as a traditional medicine as well as a food additive in eastern Asia [15]. RV can be used as an antiviral, cathartic, diaphoretic, antirheumatic, and sedative agent [16]. Scientific studies revealed that RV and its isolated compounds showed anticancer, anti-inflammatory, antioxidant, antiapoptotic, antirheumatic, antiplatelet, antimutagenic, antifibrogenic, cytoprotective, antidiabetic, and antiobesity activities [15–18]. The antiobesity action of the RV might be due to the presence of active compounds such as lutein and sulfuretin [15, 16]. Other biologically active compounds isolated from RV are fisetin, fustin, kaempferol, gallic acid, quercetin, and protocatechuic acid [15].

*Geranium thunbergii* (GT) Sieb. (Geraniaceae) is a perennial plant distributed in China, Japan, and Korea [19]. Traditionally, the whole plant has been used for diarrhea, constipation, skin diseases, and stomach ulcer. According to previous scientific reports, GT showed antimutagenic, antioxidant, anti-inflammatory, antiobesity, and BACE1 (Beta-site APP Cleaving Enzyme 1) inhibitory activities [19, 20]. Chlorogenic acid, a major phenolic compound in GT, showed antiobesity activity in high-fat diet-induced obese mice [21].

The antiadipogenic and/or antiobesity activities of *O. japonicus, R. verniciflua, G. thunbergii,* and royal jelly (RJ) were individually evaluated on the cell line, animal model, or clinical trials [10, 14, 15, 19, 22–27]. In our previous studies, we found evidence that GT, RV, OJ, and RJ showed potential antiadipogenic and/or antiobesity activity in 3T3-L1 adipocytes or Sprague-Dawley Rats [13, 16, 28–31]. Therefore, our objective was to develop an effective antiobesity herbal formulation using these natural medicines. We prepared several formulations by mixing these natural components in various ratios and prestudied their lipid inhibition activity in 3T3-L1 adipocytes. The formulation F2 was found to be most active among the prepared formulations and thus selected for further study using obese mice. In the present study, we have evaluated the antiadipogenic and antiobesity activities of F2 using 3T3-L1 adipocytes and high-fat diet (HFD) fed C57BL/6J mice. Furthermore, synergy on formulation F2 was evaluated.

## 2. Materials and Methods

### 2.1. Extraction of F2 Ingredients

*Orostachys japonicus* (OJ-aerial parts), *Rhus verniciflua* (RV-stem wood), and *Geranium thunbergii* (GT-aerial parts) were purchased from a medical herb store, Begjangseng (Iksan, Korea), and were identified by Professor Hyun-Ju Jung, Wonkwang University. Royal jelly (RJ) was obtained from Yeoju Honey Park, Korea, and was stored at −20°C until use. Lemon was purchased from a local market. The plant samples were separately extracted by reflux ing at 90 °C temperature for 3 h using ethanol solvent. The same extraction procedure was applied for plant ingredients. Before mixing the ingredients for F2, all the plant extracts, royal jelly, and lemon juice were separately dried using a freeze dryer (IIShin Lab Co., Ltd., Korea).

### 2.2. Preparation of F2

The formulation F2 was prepared by mixing a specific proportion of ethanol extracts of OJ, RV, and GT along with a trace amount of freeze-dried royal jelly and lemon juice. The ratios of each component of F2 are indicated in Table 1.

### 2.3. Phytochemical Analysis of F2 Using UPLC

An ultra-performance liquid chromatography (UPLC) system (Agilent Technologies, Santa Clara, CA, US) consisting of a G4220A 1290 Infinity Binary pump, a G4226A 1290 auto-sampler, and a G4242A 1290 DAD detector was used for the analysis of F2. The Halo C18 RP-amide column (150 mm × 2.1 mm, 2 *μ*m particle sizes) was used for the study. A suitable chromatographic solvent system consisting of (A) acetonitrile and (B) 0.5% phosphoric acid in water was optimized and performed in a gradient flow as follows: (A)/(B) = 1/99 (0 min) ⟶ (A)/(B) = 16/84 (30 min; held for 20 min). The operating conditions were as follows: flow rate, 2 mL/min; sample injection volume, 5 *μ*L; column temperature, 40°C; detection wavelength, 210 and 250 nm. Five standards, including astragalin (Kaempferol-3-O-glucoside), fustin, fisetin, sulfuretin, and ellagic acid, were used as reference compounds for the quantification of F2. For UPLC analysis, F2 and the herb ingredients were prepared at a concentration of 20 mg/mL, whereas each of the standard compounds was prepared at 1 mg/mL in methanol. All the solutions were filtered through 0.2 *μ*m PTFE hydrophilic syringe filters before injection.

### 2.4. In Vitro Antioxidant Assays

Antioxidant activity of the F2 and its ingredients was assayed by DPPH and ABTS radical scavenging activity assays. The DPPH (1,1-diphenyl-2-picrylhydrazyl) and ABTS (2,2′-azino-bis(3-ethylbenzothiazoline-6-sulfonic acid)) radical scavenging activities were assessed by using the methods described by Jeong et al. [32] and Choi et al. [33], respectively. The antioxidant activities were calculated using the following equation:

(1) $$\begin{array}{c} \text{scavenging activity}\left(\%\right)=\left[1-\left(\frac{{\text{Abs}}_{\text{sample}}}{{\text{Abs}}_{\text{control}}}\right)\right]\times 100. \end{array}$$

The results were expressed as IC_50_ (quantity of antioxidants necessary to reduce free radicals by 50% concentration). Calculations were performed using a logarithmic regression curve plotted between radical scavenging activity and treated concentrations.

### 2.5. Cell Culture and Differentiation of 3T3-L1 Adipocytes

3T3-L1 fibroblast cells (ATCC® CL-173™) were purchased from American Type Culture Collection (ATCC). The culture and differentiations of 3T3-L1 adipocytes were performed as the method described in the previous publication [30]. Briefly, the cells were grown and passaged in Dulbecco's modified eagle medium (DMEM) containing 10% newborn calf serum (NCS) and 1% penicillin/streptomycin in a humidified atmosphere of 5% CO_2_ at 37°C. The differentiation of 3T3-L1 preadipocytes was induced by treatment of the differentiating media after two days of the confluency. The differentiating media (MDI) consist of 0.5 mM methylisobutylxanthine (IBMX), 1 *μ*M dexamethasone, and 5 *μ*g/mL insulin in DMEM containing 10% fetal bovine serum (FBS). The samples were treated by mixing with MDI media. For the assessment of cell viability, the cells were treated with 5 to 80 *μ*g/mL of F2 mixing with MDI media and allowed to incubate for 8 days with changing media in alternative days. The MTT assay was done to determine cell viability on the 8^th^ day of sample treatment. The safe concentrations were treated to measure antiadipogenic activity. The synergistic antiadipogenic effect of the formulation F2 was determined by treating all the ingredients in an equivalent concentration present in 60 *μ*g/mL of F2. On the 8^th^ day of cell treatment, lipid contents accumulated in adipocytes were assessed by ORO staining.

### 2.6. RNA Extraction and Real-Time PCR Analysis

The RNA extraction, cDNA synthesis, and real-time PCR analysis were performed on 3T3-L1 adipocytes according to the previously described methods [30]. The total RNAs from the F2 treated and untreated 3T3-L1 adipocytes were extracted separately with QIAzol lysis reagent (Maryland, USA) according to the manufacturer's protocol. The cDNA was synthesized from RNA using a High-capacity RNA-to-cDNA kit (Applied Biosystems, UK) on Takara PCR Thermal Cycler (Takara, Japan). The gene expression levels were analyzed by quantitative real-time (RT) PCR. The cDNA aliquots were amplified on a StepOnePlus Real-Time PCR system from Applied Biosystems Inc. (Marsiling Industrial Estate Road 3, Singapore) using the Power SYBR-Green PCR Master Mix (Applied Biosystems, UK) in a final volume of 20 *μ*L. The primers used in the experiments were synthesized by Cosmo Genetech Co. Ltd. (South Korea) and are shown in Table 2. The *β*-actin was used as a reference gene. The relative mRNA expression levels were calculated using the ΔΔCt method [34].

### 2.7. Experimental Animals and Grouping

Male C57BL/6J mice were supplied by Central Lab. Animal Inc., Seoul, Korea. The mice were housed and allowed free access to feed and tap water under controlled and pathogen-free conditions (room temperature: 24 ± 1°C, relative humidity: 50–60%, light cycle: 7:00–19:00). The animal experiment was approved by the Wonkwang University Animal Ethics Committee (Approval No.: WKU19-78). The animals were handled in accordance with the “Guide for Care and Use of Laboratory Animals” issued by the National Institutes of Health. The mice were acclimatized to their environment for 1 week before the commencement of the experiments. Mice (age 6 weeks) were weighed and randomly divided into five groups each containing five mice. A normal group was fed with the standard chow diet (standard diet: 5L79 Orient Bio Inc., Seongnam, Korea) consisting of 13.67% fat, 20.1% protein, and 65.30% carbohydrate. The remaining four groups were fed with a high-fat diet (Rodent Diet D12451, Research Diets, New Brunswick, NJ, USA) consisting of 45% fat, 20% protein, and 35% carbohydrate. The normal and high-fat diet-fed control (HFD control) groups were treated with vehicle (0.2% carboxymethyl cellulose/PBS) orally. Two groups, F2-23 and F2-46, received F2 at doses of 23 and 46 mg/kg, twice daily, respectively, by oral gavage. A well-accepted antiobesity agent, *Garcinia cambogia* (GC), at 200 mg/kg, twice daily, was used as a positive control group. The mice were treated with respective samples from the same day of switching chow diet to HFD and continued for 8 weeks. Body weight and food intake were recorded every week. The food efficiency ratio (FER) was calculated as follows [10]:

(2) $$\begin{array}{c} \text{FER}\%=\frac{\text{gained body weight}\left(\text{g}\right)\times 100}{\text{food intake}\left(\text{g}\right)\text{during the experiment period}}. \end{array}$$

Mice feces were collected before one day of sacrifice to measure excreted fat. At the end of the experimental period, the mice were fasted for 14 hours prior to sacrifice. Blood glucose was measured before sacrifice by using a one-touch blood glucose monitoring system (CareSens ®N, i-sens, Korea). After sacrifice, the weights of epididymal adipose tissue and other organs (liver, kidney, and spleen) were determined and histological evaluation was done for liver and adipose tissue.

### 2.8. Measurement of Lipid Content in Feces

The lipid excretions into feces were extracted by using the method described by Folch et al. [35] with slight modifications. Briefly, about 0.5 g of dry feces was weighed into 15 ml polypropylene conical tubes, 3 mL of deionized water was added, and kept for 2 hours. Then feces mixture was homogenized by vortexing. Then, the feces sample was mixed with methanol : chloroform (1 : 2, v : v) and vortexed for a few minutes. The sample was then centrifuged at 1600*g* (or 2900 rpm) for 15 min. The lower lipophilic layer was collected by using a syringe, filtered, and allowed to evaporate and lipid content was measured.

### 2.9. Measurement of Tissue Weight and Histological Observation

Liver, epididymal white adipose tissues (WAT), kidney, and spleen were isolated from the mice, washed with PBS, and weighed. The liver and WAT were immediately fixed into 10% neutral formalin solution for 2 days. With alcohol dehydration, the water trapped in the tissues was removed, and then embedding was done using paraffin. All tissues were sliced to 5 *μ*m in thickness and stained with H&E (hematoxylin and eosin). The slides were examined using a light microscope and images were captured on a Canon power shot A640 camera. Images of WAT were analyzed using ImageJ software to evaluate the diameters of the adipocytes.

### 2.10. Statistical Analysis

Statistically significant differences between groups were determined using a one-way analysis of variance (ANOVA) followed by Dunnett's multiple range tests using GraphPad Prism 4 software. Data are presented as the mean ± standard deviation (SD). A *p* value of <0.05 was considered to represent statistically significant differences within groups.

## 3. Results

### 3.1. UPLC Analysis of F2

**Figure 1:**
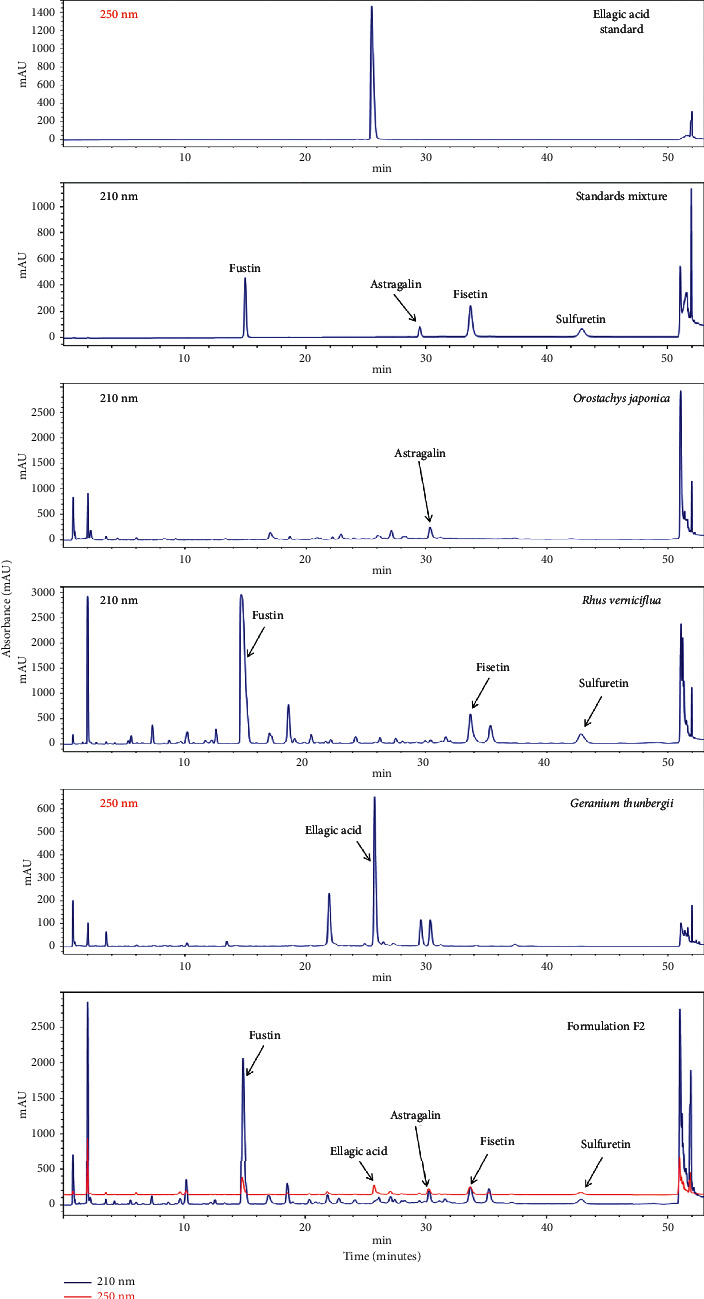
UPLC chromatograms of ellagic acid, standard mixture (astragalin, fustin, fisetin, sulfuretin), F2 components (*Orostachys japonica*, *Rhus verniciflua*, and *Geranium thunbergii*), and formulation F2.

The typical UPLC chromatograms of the standard mixture, individual plant ingredients, and F2 are shown in Figure 1. Astragalin, fustin, fisetin, and sulfuretin were detected at 210 nm, whereas ellagic acid was detected at 250 nm. The retention times of astragalin, fustin, fisetin, sulfuretin, and ellagic acid were 29.445, 14.792, 33.657, 42.800, and 25.718 min, and the concentrations of these compounds in F2 were 3.64, 35.83, 5.90, 3.22, and 1.82 mg/g, respectively.

### 3.2. Antioxidant Activity of F2

The antioxidant activities of F2 along with its components in the formulation are expressed in terms of IC_50_ in Table 3. The IC_50_ values are paralleled with standard gallic acid as reference. The result showed that the F2 showed good synergistic antioxidant activity in both DPPH and ABTS antioxidant assay models. The IC_50_ value of F2 was found to be 9.52 *μ*g/mL, which was less than OJ and GT in the formulation.

### 3.3. Effect of F2 on Cell Viability and Lipid Accumulation in 3T3-L1 Adipocytes

**Figure 2:**
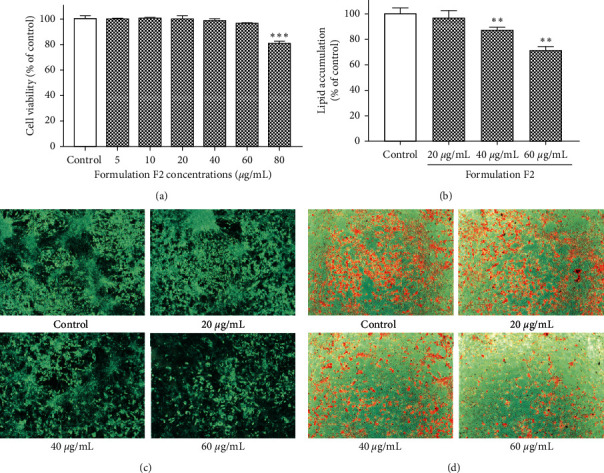
Effect of F2 on cell viability and lipid production in 3T3-L1 adipocytes. The 3T3-L1 preadipocytes were treated with F2 and cell viability (a) was assessed using MTT assay. The amount of lipid accumulation (b) on 3T3-L1 adipocytes was measured by Oil Red O (ORO) assay. The lipid accumulating cells were visualized in 10X magnification before (c) and after (d) the ORO staining. The data shown are presented as means ± SD of four separate experiments. Statistical significance was calculated using one-way ANOVA followed by Dunnett's multiple comparisons test. *∗∗p* < 0.01 and *∗∗∗p* < 0.001 vs. control.

The effect of F2 on 3T3-L1 cell viability was determined by MTT assay. The cells were treated with 5 to 80 *μ*g/mL of F2. As shown in Figure 2(a), the F2 was found to be safe below the concentration of 60 *μ*g/mL. The safe concentrations of F2 were treated for the assessment of antiadipogenic activity. Lipid accumulations in the adipocytes were visualized and quantified by the ORO staining method. F2 showed a prominent inhibitory activity on the differentiation of 3T3-L1 preadipocytes in a concentration-dependent manner (Figure 2(b)). 20, 40, and 60 *μ*g/mL of F2 were found to inhibit lipid production by 3.63 ± 6.10, 13.01 ± 2.29, and 29.13 ± 2.93 percentage, respectively, relative to blank treated control. The physical conditions of the cells were visualized before and after the ORO staining and were photographed using a light microscope (Figures 2(c) and 2(d)).

### 3.4. Synergistic Antiadipogenic Effect of F2 on 3T3-L1 Adipocytes

The highest concentration of F2 treated in 3T3-L1 adipocytes was 60 *μ*g/mL. The amount of individual ingredients presented in that concentration of F2 was calculated according to the ratio of the ingredients in the formulation (Table 1). The amount of GT, RV, OJ, RJ, and lemon equivalent to 60 *μ*g/mL of F2 was 7.481, 22.44, 29.93, 0.074 and 0.074 *μ*g/mL, respectively. To evaluate the synergistic activity, 60 *μ*g/mL of F2 and the equivalent concentration of its ingredients were separately treated. The percentage of inhibition of lipid accumulation by F2 and its ingredients is shown in Table 4. From the result, it may be estimated that the theoretical cumulative lipid inhibition would be 5.75% (measured by calculating the sum of each ingredient's mean inhibition). But the separately treated F2 showed 26.31 ± 4.73% of lipid inhibition, indicating the best synergy of its ingredients on formulation F2.

### 3.5. Effect of F2 on Gene Expression of Adipogenic Transcription Factors in 3T3-L1 Adipocytes

**Figure 3:**
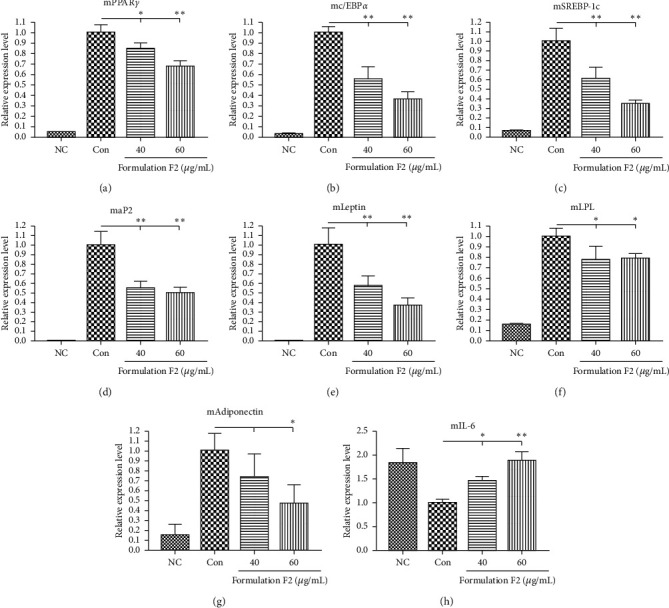
Effects of F2 on the gene expression level of adipogenic transcription factors during adipocyte differentiation. (a) Peroxisome proliferator-activated receptor gamma (PPAR*γ*), (b) CAAT/enhancer-binding protein alpha (C/EBP*α*), (c) sterol response element-binding protein-1c (SREBP-1c), (d) adipocyte protein 2 (aP2), (e) leptin, (f) lipoprotein lipase (LPL), (g) adiponectin, and (h) interleukin-6 (IL-6). Each gene's expression levels were quantified and normalized to *β*-actin. The data shown are presented as means ± SD of triplicate experiments. Statistical significance was calculated using one-way ANOVA followed by Dunnett's multiple comparisons test. *∗p* < 0.05 and *∗∗p* < 0.01 vs. control. NC: negative control (undifferentiated); Con: control (MDI).

The effect of F2 on the gene expression level of adipogenic transcription factors was examined by real-time PCR. The 3T3-L1 adipocytes were treated with 40 and 60 *μ*g/mL concentrations of F2 and total mRNA was extracted at day 8 of the sample treatment. The mRNA expression level of specific adipogenic markers (PPAR*γ*, C/EBP*α*, SREBP-1c, aP2, leptin, LPL, and adiponectin) and inflammatory marker (IL-6) was determined. As shown in Figure 3, the gene expressions of the aforementioned transcription factors and adipogenic markers were markedly downregulated whereas the expression of an IL-6 was upregulated by F2 in a concentration-dependent manner compared to the control.

### 3.6. Effect of F2 on Mice Body Weight, Food Intake, and Food Efficiency Ratio during 8 Weeks

**Figure 4:**
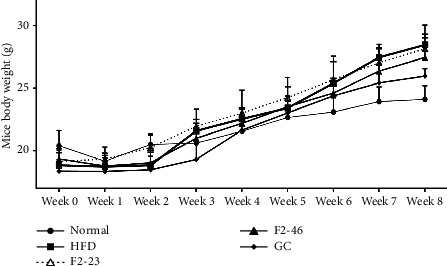
Patterns of body weight gain observed in mice during 8 weeks. Mice body weights were measured every week. Values are expressed as mean ± SD (*n* = 5). Normal: standard diet; HFD: high-fat diet control; F2-23: F2 (23 mg/kg); F2-46: F2 (46 mg/kg); GC: *Garcinia cambogia* (200 mg/kg).

The mice were treated for 8 weeks. Mice body weight gaining pattern over the treatment periods is shown in Figure 4. As presented in Table 5, the HFD control group significantly gained body weight compared with that of the normal group. However, weights of mice treated with high dose (46 mg/kg) of F2 and GC (200 mg/kg) were found to be significantly reduced within 8 weeks in comparison to the HFD control group. The food intake pattern was similar among the HFD fed groups. Food efficiency ratio (FER) for F2 (46 mg/kg) and GC was found to be lowered in comparison to the HFD control group indicating the lowest food was utilized for weight gain in those groups.

### 3.7. Effect of F2 on Lipid Excretion

**Figure 5:**
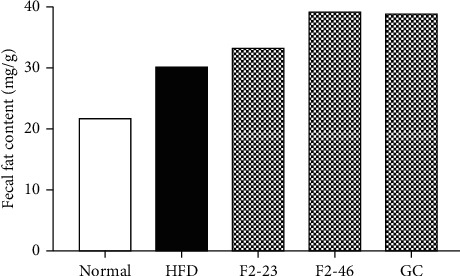
Effect of F2 on lipid excretion. Feces were collected from each group cage and lipid contents were measured collectively. The fat content was extracted with methanol-chloroform solvent and presented as percentage of dry stool weight. Normal: standard diet; HFD: high-fat diet control; F2-23: F2 (23 mg/kg); F2-46: F2 (46 mg/kg); GC: *Garcinia cambogia* (200 mg/kg).

The mice feces were collected before one day of sacrifice for the measurement of lipid content. The feces lipid content represents the excretion of dietary fat without absorption from the alimentary canal. In the current experiment, F2 and GC were found to enhance lipid excretion in feces compared with the HFD (Figure 5). The lipid excretion by F2 was increased in a dose-dependent manner. This result suggests that the reduction of body weight in the F2 group might be due to inhibition of lipid absorption in the gastrointestinal tract.

### 3.8. Effect of F2 on Fasting Blood Glucose

**Figure 6:**
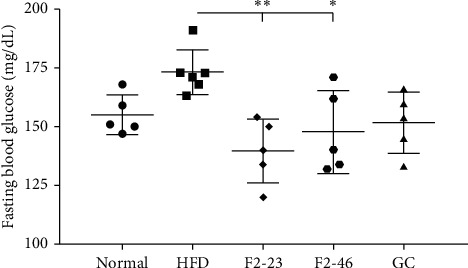
Effect of F2 on fasting blood glucose. Mice were kept fasting overnight. The levels of blood glucose were measured before mice sacrifice. Values are expressed as mean ± SD (*n* = 5). Statistical significance was calculated using one-way ANOVA followed by Dunnett's multiple comparisons test. *∗p* < 0.05 and *∗∗p* < 0.01 vs. HFD. Normal: standard diet; HFD: high-fat diet control; F2-23: F2 (23 mg/kg); F2-46: F2 (46 mg/kg); GC: *Garcinia cambogia* (200 mg/kg).

The fasting blood glucose of mice was measured before sacrifice. As shown in Figure 6, the blood glucose was elevated in the HFD control group (173.17 ± 9.52 mg/dL) compared to the normal group (155.00 ± 8.51 mg/dL). The blood glucose was significantly reduced in F2 treated groups. 23 mg/kg and 46 mg/kg treated groups showed blood glucose level of 139.60 ± 13.52 mg/dL and 147.80 ± 17.61 mg/dL, respectively. But the GC treated groups remained nonsignificant with the HFD control. The blood-glucose-lowering effect of F2 may have a beneficial effect on the management of diabetes and its complications.

### 3.9. Effect of F2 on Organ Weights and Histological Observation

**Figure 7:**
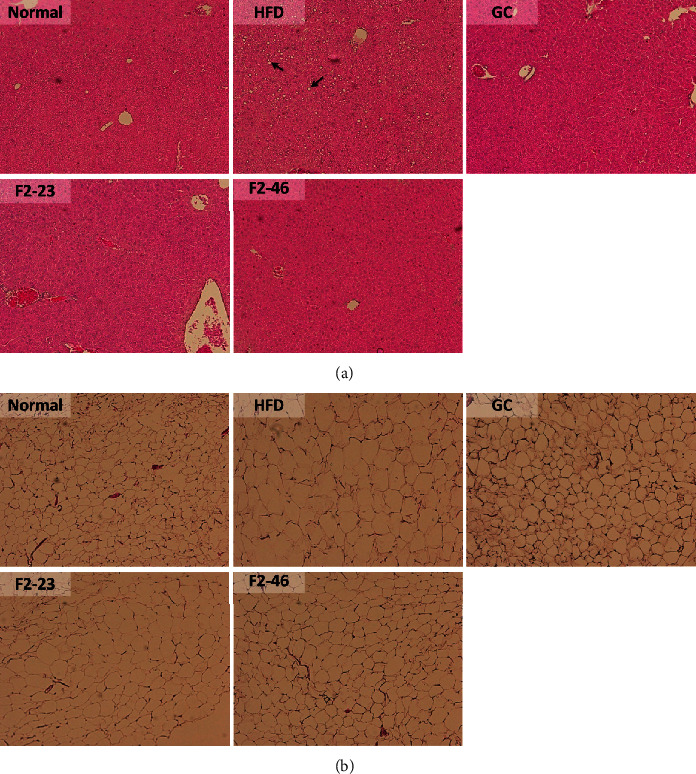
Effect of F2 on liver and white adipose tissue (WAT) histology. Histological evaluation of (a) liver and (b) WAT was done by H&E staining (magnification ×10). Lipid deposition into the liver and size of adipocytes in WAT were evaluated. The fat droplets deposited in the liver (arrows) are more noticeable in HFD control group mice. Normal: standard diet; HFD: high-fat diet control; F2-23: F2 (23 mg/kg); F2-46: F2 (46 mg/kg); GC: *Garcinia cambogia* (200 mg/kg).

**Figure 8:**
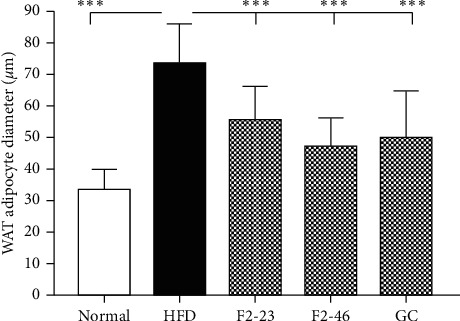
Effect of F2 on size of adipocytes in the epididymal white adipose tissue (WAT). The images of WATs from H&E staining (magnification ×10) were analyzed and diameters of the adipocytes were evaluated using ImageJ software. Results are presented as the mean ± standard deviation. Statistical significance was calculated using one-way ANOVA followed by Dunnett's multiple comparisons test. *∗∗∗p* < 0.001 vs. HFD group. Normal: standard diet; HFD: high-fat diet control; F2-23: F2 (23 mg/kg); F2-46: F2 (46 mg/kg); GC: *Garcinia cambogia* (200 mg/kg).

The epididymal white adipose tissue (WAT), liver, kidney, and spleen were isolated and weight was taken in situ. As presented in Table 6, the weights of WAT were increased significantly in the HFD group compared with that in the normal group. However, WAT weight was markedly decreased by F2 in a dose-dependent manner. Moreover, the histological morphology of epididymal WAT and liver was determined by H&E staining. The histological observation of liver tissue revealed the accumulation of lipid droplets into the liver in the HFD control group. Similarly, the sizes of adipocytes in WAT were enlarged. Treatment with F2 inhibited lipid deposition into the liver and reduced the size of adipocytes in WAT (Figure 7). The diameter of adipocytes in the WAT was significantly reduced with the treatment of F2 and GC in comparison to the HFD control (Figure 8).

## 4. Discussion

Obesity, caused by the over deposition of lipids into WAT, is perceived as a global health risk in present days. Multiple metabolic maladies such as diabetes, cardiovascular diseases, fatty liver disease, mental disorders, and even certain cancers resulting from a complication of obesity are seriously threatening human health [2, 36]. Secondarily to the dietary restriction and exercise, several drug therapies are also recommended for the management of obesity. However, unexpected adverse effects such as cardiovascular, gastrointestinal, and psychological effects associated with most of the antiobesity drugs limit their use in the general population [7]. Therefore, developing alternative therapies for obesity with minimal adverse effects is warranted. Due to having the aforementioned adverse effect along with high cost and physical dependency by long-term use of the pharmaceutical agents, plant-based remedies are gaining attention globally for the management of obesity and overweight [8]. The present study was designed to develop a novel, safe, and effective herbal formulation for the management of obesity. We developed a polyherbal formulation (F2) by homogeneously mixing of trace amount of royal jelly and lemon juice with ethanol extracts of *Orostachys japonicus* (OJ)*, Rhus verniciflua* (RV), and *Geranium thunbergii* (GT). We assessed the antiobesity efficacy of the developed formulation in 3T3-L1 adipocytes and high-fat diet-fed C57BL/6J mice. The F2 was further analyzed using UPLC and quantification was done for its major five marker compounds (astragalin, fustin, fisetin, sulfuretin, and ellagic acid).

Free radicals or reactive oxygen species are generated in the body through multiple mechanisms. Chronic inflammation associated with obesity is one of the major causes of systemic oxidative stress in the body [37], which results in the development of metabolic disorders such as insulin resistance, hypertension, asthma, etc. [38]. Herbal remedies with antioxidant potentials can counteract such obesity-associated oxidative damage and prevent comorbidities. The F2 with synergistic antioxidant activity could be a better alternative for preventing and protecting the body from obesity and related complications.

A commitment of stem cell and terminal differentiation of adipocytes into a fat-storing mature adipose cell is a crucial step in obesity [39]. Being a reliable cellular model to study adipogenesis and antiadipogenic activity [40], we selected 3T3-L1 fibroblast cells for the measurement of the antiadipogenic activity of F2. Also, most of the physiology of 3T3-L1 preadipocytes differentiation resembles that in animal tissues making it possible to extrapolate results to humans [41]. The results revealed that F2 treatment significantly reduced the lipid accumulation in 3T3-L1 adipocytes, demonstrating the ability of F2 to inhibit adipocyte differentiation (Figure 2). Previous reports mentioned that the extracts or isolated compounds from *O. japonicus* [14], *R. verniciflua* [15, 16, 26, 31], and royal jelly [30] had the antiadipogenic effect in 3T3-L1 adipocytes. The present study revealed the synergistic antiadipogenic activity of these ingredients treated even in a very small concentration in formulation F2 (Table 4).

Differentiation of preadipocytes into mature adipocytes is guided by various biochemical regulators such as insulin, expressions of adipogenic genes (PPAR*γ*, C/EBP*α*, SREBP-1c, aP2, leptin, LPL, FAS, adiponectin, etc.), and accumulations of TG and free fatty acids in cells [39, 42, 43]. Therefore, downregulation of the adipogenic transcription factors may restrict initial as well as terminal differentiation, leading to inhibition of lipid accumulation in adipocytes. In the present study, our observations revealed that the gene expression level of the adipogenic factors was significantly downregulated suggesting the crucial role of F2 in the restriction of adipogenesis (Figure 3). In another way, it is understood that the expression of pro-inflammatory cytokines like interleukin-6 (IL-6) is greater in preadipocytes than in 3T3-L1 adipocytes [44]. Hence, overexpression on mRNA level of IL-6 in F2 treated adipocytes suggested that the differentiation of 3T3-L1 preadipocyte was inhibited by F2. But the IL-6 expression was downregulated by *O. japonicas* in inflammatory human THP-1 cells [45]. Also, *R. verniciflua* decreased the level of hepatic IL-6 in LPS induced rats [46]. Other scientific reports also declared that the F2 ingredients employ potential anti-inflammatory activity [47–50]. In our study, treatment of F2 on LPS induced RAW264.7 macrophages revealed significant inhibition of nitrite production (Supplementary data, Figure S1), indicating the anti-inflammatory response of F2. Hence, the IL-6 expression upregulating effect of F2 in 3T3-L1 adipocyte might only be associated with inhibition of adipocyte differentiation but not inflammatory action. All those observations confirm the potential of the antiadipogenic and anti-inflammatory effects of F2.

The exciting outcomes from in vitro experiments encouraged us to conduct an in vivo antiobesity study. In order to confirm the potential of F2 for preventing obesity, C57BL/6J mice were fed with a high-fat diet to induce obesity and the F2 was treated simultaneously. A well-accepted antiobesity agent, *Garcinia cambogia*, was used for standard comparison. Several scientific pieces of the literature demonstrated that a high-fat diet induces obesity and diabetes in animals [51–53]. Obesity in animals can be measured by considering some criteria such as body weight gain, body fat content, and food efficiency [51, 54]. The body weight gain has been amplified in the HFD group while F2 slowed down the level of weight gaining. Previous studies revealed that ethyl acetate fraction of *O. japonicus* and 70% ethanol extract of *G. thunbergii* showed better antiobesity activity in high-fat diet-induced rodents [13, 19]. During the process of digestion, dietary fat gets absorbed from the intestine once it is subjected to emulsification with pancreatic lipase [55]. Therefore, alteration of pancreatic lipase may decrease absorption of intestinal fat. Food efficiency ratio (FER) is considered as the relation between total food intake and the amount available for anabolism to increase body weight [56]. The lower FER in F2 treated mice was due to lower weight gain on a similar amount of food consumption. This observation suggests that F2 may impair dietary fat absorption by altering pancreatic lipase. Elevation of lipid excretion in feces by F2 also supports this finding.

Hyperglycemia or diabetes is one of the major complications of obesity. Previous studies also revealed that the HFD fed mice developed hyperglycemia as well as insulin resistance [51, 57]. A high-fat diet causes a decrease in glucose transporter, insulin receptors, and glucose metabolism, and reduction in glycogen synthesis in the liver and muscle, which results in elevation of blood glucose [51]. The decreased level of fasting blood glucose by F2 in comparison to the HFD control indicates that the F2 might have a protecting effect against insulin resistance and related complications. A previous study showed that the 80% ethanol extract of *O. japonicus* was effective in lowering blood glucose in streptozotocin-induced diabetic rats [10]. Higher blood glucose level is also a consequence of lower storage of glucose in the form of glycogen due to reduction of glycogen synthase in liver and muscle tissue [58]. Besides, the reduction of glycogen synthase accelerates hepatic insulin resistance and liver steatosis, and vice versa. Accumulation of lipid components in the liver activates diacyl glycerol activated protein kinase (PKC*ε*), which further impairs the activation of insulin receptors and insulin-stimulated glycogen synthesis [59, 60]. Previous studies already exposed the scientific evidence that dietary high fat can lead to liver steatosis which is associated with obesity and liver dysfunction [61]. In our study, feeding a high-fat diet to mice for 8 weeks led to the accumulation of lipid globules in the liver causing liver steatosis in the HFD control group. However, F2 was effective in protecting the liver from steatosis (Figure 7). Obesity is primarily associated with the accumulation of lipids into white adipose tissues [62]. The visceral fat accumulation in epididymal adipocytes in the HFD control groups resulted in a significant increase in the white adipose tissue (WAT) weight. In our observations, F2 significantly reduced the tissue weight (Table 6) as well as adipocytes size (Figure 8) of WAT in dose-dependent manner supporting the effectiveness of F2 to prevent obesity in C57BL/6J mice.

## 5. Conclusions

The present study demonstrated the synergistic antiadipogenic activity of F2 in 3T3-L1 adipocytes. The reduction of adipogenesis might be a result of the downregulation of various transcription factors and adipogenic markers in the adipocytes. Further treatment of F2 in high-fat diet-fed C57BL/6J mice confirmed its preventing effect on obesity and its comorbidities. From the overall results, it may be concluded that the mechanisms of antiobesity activity of F2 include inhibition of adipocyte differentiation, restriction of dietary fat absorption, and reduction of free fatty acids accumulation in tissues. Furthermore, F2 showed potential antioxidant activity, which may alleviate obesity-related oxidative stress. In addition, F2 exhibited potent blood glucose reducing activity in high-fat diet-induced obese mice. Therefore, the novel polyherbal formulation (F2) can be a potential medication for the management of obesity and its comorbidities without adverse effects.

## Figures and Tables

**Table 1:** Composition of F2.

Ingredients	Part used	Parts by weight (%, w/w)
*Orostachys japonicus* (OJ)	Aerial parts	400 (49.88%)
*Rhus verniciflua* (RV)	Stem wood	300 (37.41%)
*Geranium thunbergii* (GT)	Aerial parts	100 (12.47%)
Royal jelly (RJ)	Whole jelly	1 (0.12%)
*Citrus limon* (Lemon)	Juice	1 (0.12%)

**Table 2:** The primer sequence used for real-time PCR.

Gene	Primer sequence (5′-3′)
PPAR*γ*	F: GTG AAG CCC ATC GAG GAC A R: TGG AGC ACC TTG GCG AAC A
C/EBP*α*	F: GCG GGA ACG CAA CAA CAT C R: GTC ACT GGT CAA CTC CAG CAC
aP2	F: AGG CTC ATA GCA CCC TCC TGT G R: CAG GTT CCC ACA AAG GCA TCA C
Leptin	F: GCC AGG CTG CCA GAA TTG R: CTG CCC CCC AGT TTG ATG
SREBP 1c	F: GGT TTT GAA CGA CAT CGA AGA R: CGG GAA GTC ACT GTC TTG GT
LPL	F: TGT AAC AAT CTG GGC TAT GAG ATC AAC R: TGC TTG CCA TCC TCA GTC CC
Adiponectin	F: GGT GCT GGG AAT TGA ACT CA R: CCT GTT TCC AGG CTT TGG CC
IL-6	F: AAA TTC GGT ACA TCC TCG ACG G R: GGA AGG TTC AGG TTG TTT TCT GC
*β*-Actin	F: GTG ACG TTG ACA TCC GTA AAG A R: GCC GGA CTC ATC GTA CTC C

PPAR*γ*, peroxisome proliferator-activated receptor-gamma; C/EBP*α*, CAAT/enhancer binding protein alpha; SREBP-1c, sterol response element binding protein-1c; ap2, adipocyte protein 2; LPL, lipoprotein lipase; IL-6, interleukin-6.

**Table 3:** IC_50_ values for the antioxidant activities of F2 and its ingredients.

Samples	DPPH-IC_50_ (*μ*g/mL)	ABTS-IC_50_ (*μ*g/mL)
Formulation F2	5.99	9.52
*Orostachys japonicus*	11.37	18.1
*Geranium thunbergii*	9.57	24.35
*Rhus verniciflua*	1.84	7.98
Royal jelly	No activity	No activity
*Citrus limon* (lemon)	No activity	No activity
Gallic acid	0.04	2.84

The percentage of DPPH and ABTS radical scavenging offered by F2, its components, and standard gallic acid plotted in logarithmic regression curve against concentration and the IC_50_ was determined by interpolation from the logarithmic regression. The antioxidant activity is expressed in the form of IC_50_ value.

**Table 4:** Synergistic action of F2 on fat reduction in 3T3-L1cells.

F2 and ingredients	Amount of ingredients in 60 *μ*g/mL of F2	Lipid inhibition (%)
Control	—	0.00 ± 3.08
GT	7.481 *μ*g/ml	5.41 ± 6.59
RV	22.44 *μ*g/ml	14.01 ± 2.36
OJ	29.93 *μ*g/ml	−7.57 ± 5.96
RJ	0.074 *μ*g/ml	−4.27 ± 4.58
Lemon	0.074 *μ*g/ml	−1.83 ± 3.93
F2	60 *μ*g/ml	26.31 ± 4.73
Cumulative inhibition of lipid production by the ingredients of F2 = 5.75%		

The synergistic effect of F2 by its ingredients on lipid production by 3T3-L1 adipocytes was determined by ORO assay. The lipid accumulations (expressed as the percentage of control) are presented as means ± SD of triplicate experiments. GT: *Geranium thunbergii*; RV: *Rhus verniciflua*; OJ: *Orostachys japonicus*; RJ: Royal jelly.

**Table 5:** Effect of F2 on mice body weight (initial and final), weight gain, food intake, and food efficiency ratio (FER) of different groups during 8 weeks of the experiment.

Groups	Body weight (g)		Weight gain (g)	Food intake (g/mice)	FER (%)
	Initial	Final			
Normal	20.40 ± 1.21	24.10 ± 1.10	3.79 ± 0.68*∗∗∗*	180.08	2.11 ± 0.38*∗∗∗*
HFD	18.85 ± 0.99	28.46 ± 0.84	9.61 ± 0.56	113.3	8.48 ± 0.51
F2-23	19.16 ± 0.90	28.15 ± 1.88	8.99 ± 1.19	117.85	7.63 ± 1.01
F2-46	19.35 ± 0.83	27.46 ± 1.57	8.11 ± 0.79*∗*	120.25	6.75 ± 0.65*∗∗*
GC	18.37 ± 0.93	25.96 ± 0.60	7.586 ± 0.79*∗∗*	110.23	6.88 ± 0.72*∗∗*

Food intake and body weight gains were measured every week. Food efficiency ratio (FER) was calculated as follows: FER% = gained body weight (g) × 100/food intake (g). Results are presented as the mean ± SD (*n* = 5). Statistical significance was calculated using one-way ANOVA followed by Dunnett's multiple comparisons test. *∗p* < 0.05, *∗∗p* < 0.01, and *∗∗∗p* < 0.001 vs. HFD group. Normal: standard diet; HFD: high-fat diet control; F2-23: F2 (23 mg/kg); F2-46: F2 (46 mg/kg); GC: *Garcinia cambogia* (200 mg/kg).

**Table 6:** Effect of F2 on organ weight.

Groups	WAT (g)	Liver (g)	Kidney (g)	Spleen (mg)
Normal	0.46 ± 0.09*∗∗∗*	0.94 ± 0.05*∗*	0.29 ± 0.02	53.17 ± 5.71
HFD	1.38 ± 0.09	0.82 ± 0.08	0.28 ± 0.02	60.59 ± 8.99
F2-23	1.10 ± 0.32*∗*	0.75 ± 0.07	0.29 ± 0.02	59.50 ± 5.32
F2-46	0.75 ± 0.09*∗∗∗*	0.80 ± 0.06	0.30 ± 0.03	63.30 ± 5.68
GC	0.92 ± 0.15*∗∗*	0.77 ± 0.08	0.30 ± 0.02	57.88 ± 10.49

Results are presented as the mean ± standard deviation (*n* = 5). Statistical significance was calculated using one-way ANOVA followed by Dunnett's multiple comparisons test. *∗p* < 0.05, *∗∗p* < 0.01, and *∗∗∗p* < 0.001 vs. HFD group. Normal: standard diet; HFD: high-fat diet control; F2-23: F2 (23 mg/kg); F2-46: F2 (46 mg/kg); GC: *Garcinia cambogia* (200 mg/kg).

## Data Availability

The data will be made available upon reasonable request to the corresponding author.

## Supplementary Materials

Supplementary Materials In vitro anti-inflammatory activity of F2 on RAW264.7 macrophage: the Raw264.7 macrophages (ATCC® TIB-71™), used to determine the anti-inflammatory efficacy of F2, were purchased from American Type Culture Collection (ATCC). The macrophages were induced by lipopolysaccharide (LPS) and different concentrations of F2 were treated. The absorbance of nitrites produced by LPS induced RAW264.7 macrophages was measured spectrometrically and the amount was calculated using linear regression curve of sodium nitrite standard. The result showed that nitrite production was significantly higher in LPS induced control wells compared to the noninduced control. The treatments of F2 at the concentration of 20, 40, and 80 *μ*g/mL significantly reduced LPS nitrite production. This result suggested that the F2 may exhibit anti-inflammatory activity.
