# Supplementary material for: Efficacy of a Novel Herbal Formulation (F2) on the Management of Obesity: In Vitro and In Vivo Study
Source: Evid Based Complement Alternat Med. 2021 Feb 8;2021:8854915. doi: 10.1155/2021/8854915 (PMC7884115; doi:10.1155/2021/8854915)
Supplement: Supplementary Materials — In vitro anti-inflammatory activity of F2 on RAW264.7 macrophage: the Raw264.7 macrophages (ATCC® TIB-71™), used to determine the anti-inflammatory efficacy of F2, were purchased from American Type Culture Collection (ATCC). The macrophages were induced by lipopolysaccharide (LPS) and different concentrations of F2 were treated. The absorbance of nitrites produced by LPS induced RAW264.7 macrophages was measured spectrometrically and the amount was calculated using linear regression curve of sodium nitrite standard. The result showed that nitrite production was significantly higher in LPS induced control wells compared to the noninduced control. The treatments of F2 at the concentration of 20, 40, and 80 μg/mL significantly reduced LPS nitrite production. This result suggested that the F2 may exhibit anti-inflammatory activity. [file 8854915.f1.docx]

*Supplementary Materials*

Efficacy of a novel herbal formulation (F2) on the management of obesity: *in vitro* and *in vivo* study

Prakash Raj Pandeya^1^, Ramakanta Lamichhane^1^, Kyung-Hee Lee^1^, Gopal Lamichhane^1^, Se-Gun Kim ^2^ and Hyun-Ju Jung^1^*

^1^ Wonkwang University, College of Pharmacy, Dept. of Oriental Pharmacy, & Wonkwang-Oriental Medicines Research Institute, Iksan, Jeonbuk 54538, Republic of Korea

^2^ Department of Agricultural Biology, National Academy of Agricultural Science, Rural Development Administration, Wanju 566-851, Republic of Korea

***** Correspondence: [hyun104@wku.ac.kr](mailto:hyun104@wku.ac.kr); [Tel.: +82-63-850-6814](Tel:+82-63-850-6814)

***In-vitro* anti-inflammatory activity of F2 on RAW264.7 macrophage**

The Raw264.7 macrophages were used to determine the anti-inflammatory efficacy of F2. The macrophages were induced by lipopolysaccharide (LPS) and different concentrations of F2 were treated. The absorbance of nitrites produced by LPS induced RAW264.7 macrophages was measured spectrometrically and the amount was calculated using a linear regression curve of sodium nitrite. The result showed that nitrite production was significantly higher in LPS induced control wells compared to the non-treated control. The treatments of F2 at the concentration of 20, 40, and 80 µg/mL were found to significantly active in reducing nitrite production. This result indicated the F2 may have anti-inflammatory activity.


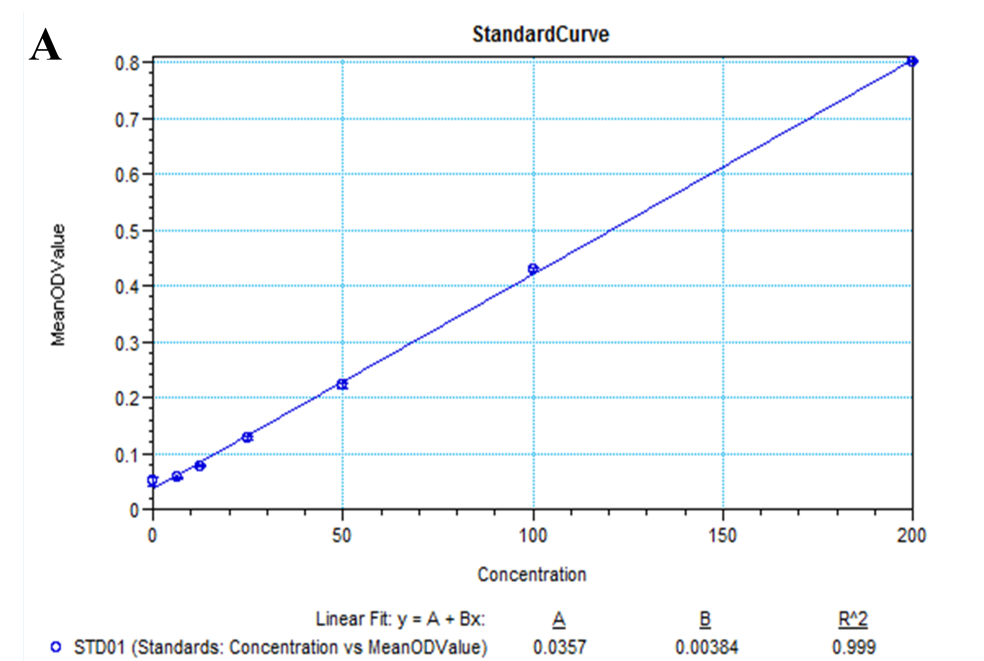


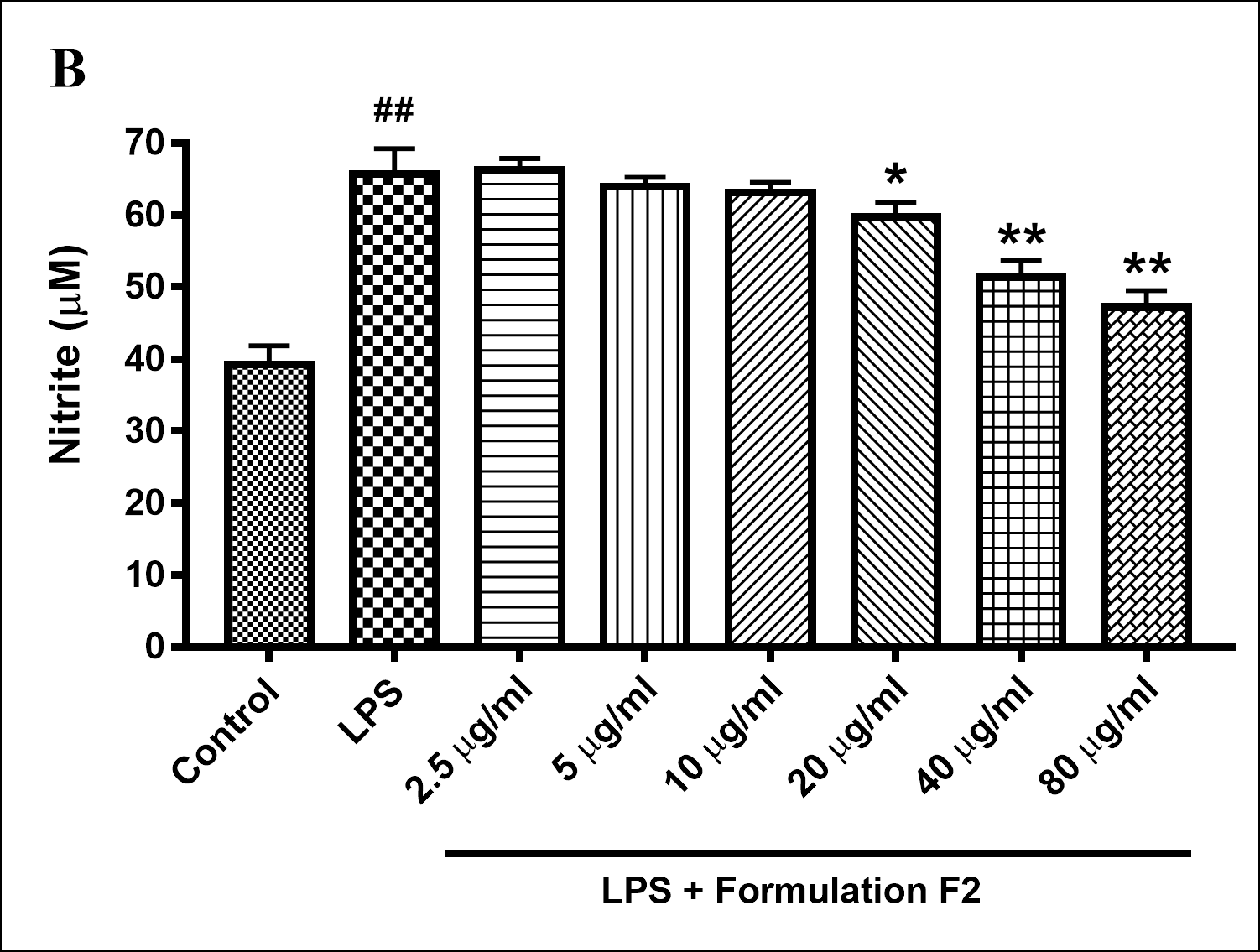


**FIGURE S1.** Effect of F2 on Nitrite production by LPS induced RAW264.7 macrophage. (A) A standard curve was plotted as the concentration of sodium nitrate versus absorbance. The amount of (B) nitrite production in LPS treated or non-treated wells were calculated using the standard curve. Values are expressed as mean ± SD. Statistical significance was calculated using one-way ANOVA followed by Dunnett’s multiple comparisons test. *P < 0.05; and **P < 0.01 vs. LPS control. ##P < 0.01 vs. LPS untreated control.
